# Teaching Bleeding Control and Building Trust With a Community Affected by Firearm Injuries

**DOI:** 10.1001/jamasurg.2024.3372

**Published:** 2024-09-11

**Authors:** Kathryn M. Stadeli, Farah B. Mohamed, Lauren L. Agoubi, Abdifatah Dahiye, Elina Serrano, Maymuna Haji-Eda, Monica S. Vavilala

**Affiliations:** 1 Department of Surgery, University of California, Davis, Sacramento; 2 Department of Surgery, University of Washington, Seattle; 3 Harborview Injury Prevention and Research Center, Seattle, Washington; 4 Global Health, University of Washington, Seattle; 5 Seattle and King County Public Health, Seattle, Washington; 6 Somali Health Board, Seattle, Washington; 7 Department of Anesthesiology, University of Washington, Seattle

## Abstract

This survey study evaluates a program for increasing bystander bleeding control skills, improving self-efficacy for bleeding control, and building trust between community participants and first responders in a Somali community in the US affected by firearm-related deaths.

Communities affected by firearm injuries have lower rates of bystander interventions^1^ and may mistrust first responders (FRs) (emergency medical services [EMS] and police).^2,3^ A pilot session of a community-engaged, culturally adapted program (Working for Equity: WE Stop the Bleed [WE STB]^2^) demonstrated promise for increasing bystander bleeding control skills, improving self-efficacy^4^ for bleeding control, and building trust between community participants (CPs) and FRs in a Somali community in Seattle, Washington, affected by high rates of firearm-related deaths. We aimed to iteratively study this program to evaluate reproducibility of results and feasibility of program expansion with new participants within this community.

## Methods

The WE STB program was implemented in person within the Seattle-area Somali community and included small group cultural exchange (Somali Health Board moderators) and the STB^5^ course (physician and FR instructors). Pilot program and conceptual model details were previously described.^2^ The Somali Health Board recruited CPs through word of mouth and social media. First responders were recruited via emails to EMS and police leadership; all trained as STB instructors. Gift cards were offered for survey completion. English and Somali languages were used, with interpreters available. Programmatic changes and survey edits for clarity were iteratively implemented each session based on feedback (eMethods in Supplement 1). Race was self-reported by FRs to see how racial identity among FRs may or may not be representative of the communities they serve; CP surveys did not include race because all participants identified as part of the Somali community. Bleeding control knowledge and self-efficacy were evaluated (CPs only) using multiple-choice pre/post surveys. Trust and comfort between CPs and FRs were evaluated (both groups) through thematic analysis of open-ended survey responses. Acceptability was evaluated through willingness to recommend the program (both groups). Statistical significance was evaluated with a 2-tailed *t* test, with *P* < .05 indicating significance. This survey study follows the AAPOR reporting guideline. The University of Washington institutional review board determined the study to be exempt because of minimal risk. Information regarding informed consent was written on the first page of the survey. Participants consented via completing the survey.

## Results

Three sessions with distinct participant groups were conducted in January 2020, June 2022, and July 2022 (95 CPs and 29 FRs [23 EMS and 6 police]). Survey response rates were 75% or greater for both groups. Participant characteristics are reported in Table 1. Community participant knowledge and self-efficacy improved from before to after the course (from 33% to 51% [*P* < .001] and from 77% to 97% [*P* < .001], respectively). Sixty-five of 67 CPs (97%) and 23 of 24 FRs (96%) would recommend the training to a friend or colleague. After course completion, 23 of 61 CPs (23%) still expressed concern they would be held responsible if they helped in an emergency and a bad outcome occurred.

**Table 1.** Self-Reported Characteristics of Community Participants and First Responders

Characteristic	Community participants (n = 95)	First responders (n = 29)
Sex		
Female	61/78 (78)	7/25 (28)
Male	17/78 (22)	18/25 (72)
Age, median (IQR), y	38 (31-42)	34 (30-43)
Race^a^		
American Indian or Alaska Native	NA	0
Asian	NA	5/24 (21)^b^
Black or African American	NA	0
Native Hawaiian or Pacific Islander	NA	2/24 (8)^b^
White	NA	19 (79)
Other	NA	0
Languages used to communicate in health care settings^c^		
Somali and English	37/82 (45)	0
Amharic and English	1/82 (1)	0
Somali and Amharic	1/82 (1)	0
Somali only	38/82 (46)	0
English only	5/82 (6)	18/25 (72)
English and Spanish	0	4/25 (16)
English and German	0	2/25 (8)
English and French	0	1/25 (4)
Speak English less than very well (limited English proficiency)	55/82 (67)	NA
Born outside the US	79/82 (95)	NA
Time in the US, median (IQR), y	13.5 (10-18)	NA
Time of first responder job experience, median (IQR), y	NA	8 (4-17)
Survey response rate		
Presurvey	86/95 (91)	25/29 (86)
Postsurvey	71/95 (75)	24/29 (83)

^a^ Community participant surveys did not include self-reported race. Community partners thought this would be inappropriate given that all participants identified as part of the Somali community and emphasized that most identify their race and origin as Somali rather than Black or African American. First responders were able to choose from the listed categories of race or write-in their own description (other).

^b^ Individuals who identified as Native Hawaiian or Pacific Islander also identified as Asian; therefore, the total is equal to more than 100%

^c^ Some participants felt comfortable using additional languages in health care settings as third or fourth languages, including Swahili, Kiswahili, Oromo, Hindi, and Urdu.

Positive qualitative changes in comfort and trust were reported by FRs and CPs (Table 2). Most FRs (N = 16) reported the small group discussion and interactions as the best part of the training; CPs (N = 12) most frequently cited the hands-on training as their favorite. Substantive iterative changes based on feedback included moving small group discussion to the beginning of the session and shortening lecture time and increasing time teaching in small groups.

**Table 2.** Key Themes From Open-Ended Surveys Responses

Theme	Counts of themes^a^		Illustrative examples
	CPs (n = 56)	FRs (n = 24)	
Positive trust and/or comfort	19	8	“It made me more comfortable being able to directly discuss my concerns [regarding police].” – CP
			“I think [interacting] was less intimidating after getting to know [the community participants].” – FR
Positive value of small group discussions and interactions	21	20	“[Small group] allows for more comfortable environment to share feeling or concerns.” – CP
			“People were more willing to speak in small groups.” – FR
Shorten lecture and/or increase small group time (hands-on training and/or discussion)	2	14	“[would change the training by] longer plus other topics were able to be discussed.” – CP
			“Maybe make a little more time for meaningful conversations.” – FR
Small group discussion and interaction was best part of the training	4	16	“[The best part was] answering our questions.” – CP
			“[The best part of the training was] getting to know and interact with the community.” – FR
Hands-on training was best part of the session	12	6	“[The best part was] the hands on. I loved being taught how to handle any emergency.” – CP
			“I think the practical was the best part for the table I worked with.” – FR
Overall Stop the Bleed training was best part of the session	9	0	“I liked the training that I can help myself and the community.” – CP

Abbreviations: CP, community participant; FR, first responder.

^a^ Data are the number of respondents who indicated each theme in their open-ended responses. Respondents who indicated a particular theme more than once were only counted once. Some respondents did not answer all questions.

## Discussion

The results of this evaluation suggest that WE STB is a feasible and acceptable approach to increase bleeding control knowledge and self-efficacy among CPs and build trust and comfort between CPs and FRs.^6^ Iterative changes, specifically shifting time from lecture to small groups and doing small group discussion before the STB course, were key to program improvement. Evaluation survey translation introduced challenges with question fidelity and understanding, which likely contributed to lower scores on knowledge questions. All sessions were conducted within 1 geographic and cultural community; some adaptations may not be appropriate for other communities. Further evaluation of the WE STB program is currently under way with diverse US communities.

## Notes

Supplement 1. **eMethods.** Additional Methodological Information

Supplement 2.
Data Sharing Statement
