# Supplementary material for: Teaching Bleeding Control and Building Trust With a Community Affected by Firearm Injuries
Source: JAMA Surg. 2024 Sep 11;159(11):1324–6. doi: 10.1001/jamasurg.2024.3372 (PMC11391356; doi:10.1001/jamasurg.2024.3372)
Supplement: Supplement 1. — eMethods. Additional Methodological Information [file jamasurg-e243372-s001.pdf]

Supplementary Online Content

Stadeli KM, Mohamed FB, Agoubi LL, et al. Teaching bleeding control and building trust with a community affected by firearm injuries. *JAMA Surg.* Published online September 11, 2022. doi:10.1001/jamasurg.2024.3372

eMethods. Additional Methodological Information

This supplementary material has been provided by the authors to give readers additional information about their work.

© 2024 Stadeli KM et al. *JAMA Surgery.*

eMethods. Additional Methodological Information

The WE Stop the Bleed program expansion was conducted for three separate sessions with three distinct participant groups over a period of 2 years. Each session was held in-person. After each session, feedback from surveys and verbal feedback from participants was compiled and iterative changes were made for the next session, including edits to wording or translation of survey questions (though questions themselves were not changed), progressively moving more of the content from lecture-style to small groups over each session, and moving the small group discussion from the end of the session for the 3rd session. The same survey questions (with edits) were asked after each session regardless of iterative changes to program implementation.

© 2024 Stadeli KM et al. *JAMA Surgery.*
