# Supplementary material for: Teaching Bleeding Control and Building Trust With a Community Affected by Firearm Injuries
Source: JAMA Surg. 2024 Sep 11;159(11):1324–6. doi: 10.1001/jamasurg.2024.3372 (PMC11391356; doi:10.1001/jamasurg.2024.3372)
Supplement: Supplement 2. — Data Sharing Statement [file jamasurg-e243372-s002.pdf]

Data Sharing Statement

Stadeli. Teaching Bleeding Control and Building Trust With a Community Affected by Firearm Injuries. *JAMA Surg*. Published September 11, 2024. doi:10.1001/jamasurg.2024.3372

Data
Data available: No
